# Is seafood dangerous for Silesian population?

**DOI:** 10.1186/2045-7022-1-S1-P109

**Published:** 2011-08-12

**Authors:** Barbara Rymarczyk, Joanna Gluck, Barbara Rogala

**Affiliations:** 1 Medical University of Silesia, Chair and Clin. Dept. of Internal Diseases, Allergology and Clin. Immunology, Katowice, Poland

## Background

Business and touristic migration strongly influence the nutritional habits and alter the allergy profile in population. The aim of the study was the assessment of nutritional habits in the population of Upper Silesia, the role of fish and/or seafood in food hypersensitivity, clinical characteristic of patients suffering from seafood hypersensitivity reactions.

## Material and methods

238 subjects (146 women, 92 men, mean age 40,95±13,31 yrs with self-reported hypersensitivity reactions after fish and/or seafood ingestion were enrolled into the study. All of them were asked to answer a few questions concerning detailed medical history, clinical symptoms of food hypersensitivity and nutritional habits. Each of them underwent skin prick tests with twelve most common food allergens including fish and seafood allergens. Allergen-specific IgE serum levels against food allergens were assessed.

## Results

Fish/seafood hypersensitivity reactions were reported in (n=18; 7,56%) cases: generalized urticaria (n=9; 50%), convincing history of anaphylaxis (n=4; 22,22%), angioedema (n=2; 11,11%), gastrointestinal disturbances (n=2; 11,11%), oral allergy syndrome (OAS) (n=1; 5,56%). 22,2% (n=4) reported fish /seafood as the only eliciting factor. 50% symptoms of hypersensitivity reactions (n=9) begun during the first hour after ingestion of the offending food, less frequently in the first 12 hours (n=7; 38,89%). Only in 2 cases (11,11%) the symptoms occurred after 12 hours. Eight patients (44,44%) had a positive family history of atopy. Serum levels of allergic specific IgE against fish and/or seafood reached 2 class (0,7-3,5 kU/l) in 3 patients (16,7%). Only 1 patient (5,56%) showed positive skin prick tests with lobster allergen.

## Conclusion

Relatively low consumption of fish and/or seafood in Upper Silesia population results in a low frequency of hypersensitivity reactions. The majority of reactions occur in a short time after ingestion. Searching for trigger factors of anaphylaxis the seafood must always be taken into account.

